# Management of Essential Thrombocythemia in a Patient With a History of Thrombotic Thrombocytopenic Purpura

**DOI:** 10.7759/cureus.57716

**Published:** 2024-04-06

**Authors:** Samuel Johnson, Albert Lee, Quayd Robertson, Stephen D Wagner

**Affiliations:** 1 Internal Medicine, Unity Health, Searcy, USA; 2 Diagnostic Radiology, University of Missouri, Columbia, USA

**Keywords:** myeloproliferative neoplasm disease, calr mutation, adamts 13, thrombotic thrombocytopenic purpura, essential thrombocythemia

## Abstract

This case presents a patient with two transposed rare diagnoses developed within 10 years. Thrombotic thrombocytopenic purpura (TTP) and essential thrombocythemia (ET) are disease processes that present with opposite clinical and laboratory findings. The patient was diagnosed with ET over a decade after the initial TTP diagnosis when she was found to have extreme thrombocytosis during routine laboratory monitoring. The patient was found to have the calreticulin (CALR) mutation variant of ET which is associated with increased platelet production and she was started on hydroxyurea and aspirin. Subsequent management of the patient’s TTP relapses and large fluctuations in her platelet counts necessitated adjustments to the standard ET treatment regimen. There is little to no literature on this rare comorbidity and further investigation is needed for the association between these diseases and modifications to standard treatment to prevent relapses and sequelae.

## Introduction

Essential thrombocythemia (ET) and thrombotic thrombocytopenic purpura (TTP) are rare hematologic disorders that both share a fluctuation in platelet counts [1]. TTP has an incidence of only a few cases per million people each year and generally causes thrombocytopenia [1]. This disease is characterized by an agglomeration of von Willebrand factor multimers that can increase thrombus formation [1]. The thrombi are due to concurrent deficiency of a disintegrin and metalloproteinase with a thrombospondin type 1 motif, protease member 13 (ADAMTS13) activity, which is a protease that cleaves von Willebrand factor [2]. This can lead to a classic pentad of microangiopathic hemolytic anemia, thrombocytopenia, fever, renal dysfunction, and neurological symptoms [3]. In contrast, ET is generally classified as a myeloproliferative neoplasm (MPN) with a coinciding increase in platelet production [3]. ET has a similar prevalence to TTP and is commonly associated with mutations in JAK2 V617F (50-55%), CALR (20-25%), and MPL (2-5%) [4,5]. Most patients with these driver mutations present asymptomatic, but many progress to having hemorrhagic, thrombotic, or vasomotor issues [3].

TTP can either be inherited (5% of cases) or acquired (95% of cases) [3]. Acquired cases are thought to have an autoimmune etiology [3]. ET is associated with a mutation in a somatic driver gene that upregulates the production of platelets [3]. There is evidence in the literature of the possibility of ET following or accompanying another autoimmune disease [6]. Our case presents a patient with a history of TTP and the subsequent presentation of ET with confirmed decreased ADAMTS13 activity and positive CALR mutation. The concurrence of ET and TTP in a patient has rarely been described in the research literature. This report describes a patient with multiple relapses of diagnosed TTP and the development of ET. This individual's ET led to significant fluctuations in her platelet count complicating her treatment regimen.

## Case presentation

A 59-year-old Caucasian female was diagnosed with TTP in 2005 after an initial presentation with neurologic symptoms of word-finding difficulty and vision changes. The patient was treated with vincristine, rituximab, intravenous immune globulin (IVIG), corticosteroids, and plasmapheresis, and subsequently went into remission. Ten years later, she presented with clinical and laboratory findings consistent with a relapse of her disease. She had multiple mild episodes of TTP recurrence prior to this presentation but this episode was a complete relapse of her symptoms. Her ADAMTS13 activity was undetectable during this visit, and her anti-ADAMTS13 IgG level was 1.7 units/ml. She was started on plasmapheresis and a standard regimen of rituximab at 375 mg/m^2^ once a week for four weeks. Her disease was followed periodically throughout the year with her ADAMTS13 activity being reported as low to normal and her assay showing undetectable antibody levels.

Two years later during a routine follow-up of her condition, the patient was found to have a platelet count of 1,239 (G/L). Her platelet count was quite alarming, and therefore, an MPN panel was run to assess for genetic mutations and the patient was diagnosed with ET with the CALR mutation variant. Due to the extreme elevation in the patient’s platelet count, a ristocetin cofactor assay was performed to evaluate for von Willibrand disease which can occur with extreme thrombocytosis, but it was negative. The patient was started on hydroxyurea 500 mg BID and low-dose aspirin.

**Figure 1:**
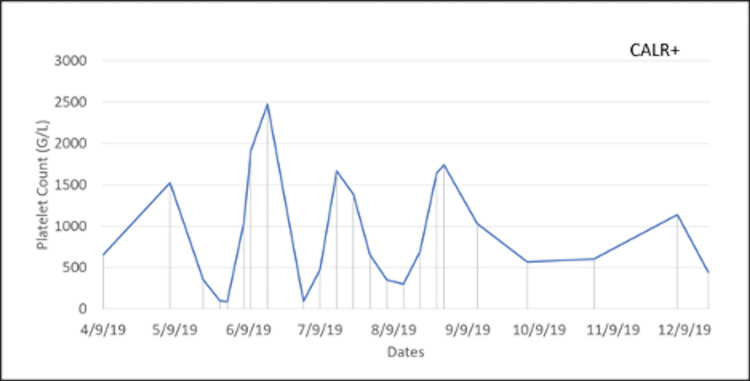
Fluctuations in the patient’s platelet counts during year one of weekly monitoring.

**Figure 2:**
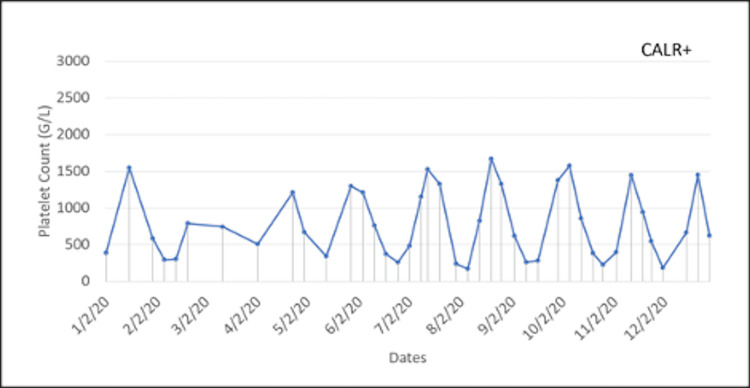
Fluctuations in the patient’s platelet counts during year two of weekly monitoring.

**Figure 3:**
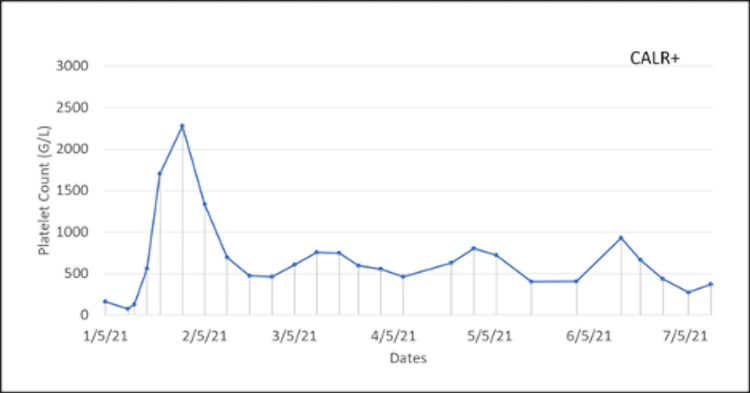
Fluctuations in the patient’s platelet counts during year three of weekly monitoring.

Two years after the ET diagnosis, she presented again with word-finding and visual disturbances like her previous relapses of TTP. Hydroxyurea and aspirin were held, and she was started on plasmapheresis and the standard regimen of rituximab. She was on and off hydroxyurea following her TTP recurrence. Once settled, she was restarted on 1500 mg/day hydroxyurea and low-dose aspirin, with the dosing being adjusted frequently due to extreme swings in her platelet count. In 2019, a bone marrow biopsy was performed to characterize her ET further and revealed variably hypercellular bone marrow for her age (70% cellularity) with megakaryocytic hyperplasia consistent with MPN. Since then the patient's ET was managed with hydroxyurea 1500 mg/day: 1,000 mg in the morning and 500 mg in the evening. Her case is rare in that her platelet count fluctuated considerably, even with her treatment. Concerned about the severe side effects associated with high and low platelet counts, the patient was seen weekly to monitor the fluctuations in her platelet count, as seen in Figures 1-3. When her platelet counts dropped below 100 (G/L), her hydroxyurea was lowered to 1,000 mg for the current day, and then she restarted her normal regimen of 1,500 mg/day.

## Discussion

This case is unique in that the occurrence of two pathological phenomena, TTP and ET, is rare and not commonly cited in the literature. Based on our literature review, we found only one other case that presented this phenomenon [1]. However, there have been cases of individuals diagnosed with immune thrombocytopenia (ITP) and the development of ET [1,2,7]. 

Some studies have reported a relationship between specific treatments, such as azathioprine, for autoimmune diseases to be the inciting factor in the development of ET [6,7]. This case brings forth another example of a patient with an autoimmune disease, her acquired TTP, and the subsequent development of ET. However, this relationship is not fully understood. Mutant CALR activates the extracellular thrombopoietin receptor (MPL) on hematopoietic cells which leads to constitutive activation of the Jak/Stat pathway causing overproduction of platelets [7]. It is postulated that autoantibodies play a role in modulating this pathway [1]. Based on this hypothesis, it is possible that antibodies may interact with mutant CALR and affect its binding to MPL. The other consideration would be the possibility of a drug interaction, but that does not appear to be the scenario in this case because the patient has not been exposed to any of the postulated drugs associated with the development of ET [7]. Further studies on the development of ET following the diagnosis of an autoimmune condition could be very beneficial in understanding the pathophysiology and pave the way for possible prevention.

The presentation of this patient’s ET was quite aggressive, with fluctuations in her platelet count occurring weekly despite appropriate and consistent treatment. Figures 1-3 show large swings in the platelet count over a few years of follow-up. This constant swing in platelet count complicated an already difficult disease process and the opposing pathology of TTP. This requires further exploration into the standard treatment for patients with ET and how a patient with a concurrent diagnosis of TTP should be managed. 

The goal of treatment of ET is to prevent thrombotic/hemorrhagic complications and to alleviate symptoms (e.g., headaches, dizziness, visual disturbances, burning dysesthesia). Available treatment options are not curative and have not been shown to prolong survival nor prevent disease transformation to acute myeloid leukemia (AML) or post-ET myelofibrosis [4]. Patients with ET are first analyzed using the International Prognostic Score of Thrombosis in the World Health Organization essential thrombocythemia (IPSET-thrombosis) score [4]. The score places a patient into a risk category and then dictates how the patient will be managed. Patients at high-to-intermediate risk are treated with a cytoreductive drug such as hydroxyurea and should be given either an antiplatelet drug or an anticoagulant [5]. Patients at a low-to-very low risk are treated with low-dose aspirin or observation alone. A significant component of risk stratification includes whether a patient has experienced any vasomotor symptoms or has a history of thrombosis. Patients with a platelet count greater than one million per microliter should also have a ristocetin assay done to test for acquired von Willebrand syndrome (aVWS) [4]. This syndrome occurs because excessive thrombocythemia can lead to the absorption of large von Willebrand multimers [4]. In patients with aVWS, aspirin is a contraindication due to the high risk of bleeding. Regardless of the risk category, a patient with platelet counts greater than one million per microliter should be on a cytoreductive agent [5]. Hydroxyurea is the most common cytoreductive agent used and dosing is initially based on the patient’s weight with subsequent titration dependent on weekly platelet levels [5]. Hydroxyurea has a rapid onset of action and rapid clearance with a half-life of two to four hours [5]. Therefore, regimen changes should be made cautiously to demonstrate a baseline [5].

This case presents a patient who fell in the high-to-intermediate risk category. She was managed on hydroxyurea 1,500 mg per day and low-dose aspirin. One of the difficulties with the patient’s treatment management was the possibility of relapse of her TTP. Therefore, she was not placed on systemic anticoagulation therapy. The patient was followed weekly by a hematologist to monitor her bloodwork. Adjustments to her hydroxyurea dosage were made when the patient's platelet counts were below 100 per microliter. The patient was instructed to reduce her hydroxyurea dose from 1,500 mg to 1,000 mg for one day and then continue her routine regimen. This adjustment was well tolerated without any ET sequelae occurring.

## Conclusions

There is much to learn about the pathophysiology associated with developing ET following the diagnosis of an autoimmune disease as seen in our case report. Here, we presented a rare case of opposing pathologies and an aggressive presentation of ET. Further investigation into optimal ET treatment and medication adjustments with a concurrent diagnosis of TTP is needed.
